# Strategies to optimize respiratory muscle function in ICU patients

**DOI:** 10.1186/s13054-016-1280-y

**Published:** 2016-04-19

**Authors:** Willem-Jan M. Schellekens, Hieronymus W. H. van Hees, Jonne Doorduin, Lisanne H. Roesthuis, Gert Jan Scheffer, Johannes G. van der Hoeven, Leo M. A. Heunks

**Affiliations:** Department of Anesthesiology, Radboud University Medical Centre, Nijmegen, 6500 HB The Netherlands; Department of Pulmonary Diseases, Radboud University Medical Centre, Nijmegen, 6500 HB The Netherlands; Department of Intensive Care Medicine, Radboud University Medical Centre, Nijmegen, 6500 HB The Netherlands

## Abstract

Respiratory muscle dysfunction may develop rapidly in critically ill ventilated patients and is associated with increased morbidity, length of intensive care unit stay, costs, and mortality. This review briefly discusses the pathophysiology of respiratory muscle dysfunction in intensive care unit patients and then focuses on strategies that prevent the development of muscle weakness or, if weakness has developed, how respiratory muscle function may be improved. We propose a simple strategy for how these can be implemented in clinical care.

## Background

Respiratory muscle weakness may develop in ventilated critically ill patients [1–4]. For instance, Jaber and colleagues [1] demonstrated that after 5–6 days of controlled mechanical ventilation in intensive care unit (ICU) patients, force-generating capacity of the diaphragm was reduced by ±32 %. In ICU patients, impaired capacity of the respiratory muscles is accompanied by an increased load due to elevated elastic and resistive forces of the respiratory system. This imbalance in load and capacity plays an important role in the development of ventilatory failure, for instance during a weaning trial. Respiratory muscle weakness is associated with adverse clinical outcomes, including difficult weaning from mechanical ventilation, increased mortality, and increased risk of ICU/hospital readmission [5]. It is reasonable to propose that strategies that aim to restore respiratory muscle function in these patients improve outcome. The aim of this review is to discuss (future) strategies that prevent the development of respiratory muscle weakness or restore respiratory muscle function in weak ICU patients. We will mainly focus on interventions that are most likely to be of clinical importance in the near future.

## Pathophysiology of respiratory muscle weakness in the critically ill

Reduced force output of the respiratory muscles in the critically ill may result from injury at any point between the central respiratory centers and the contractile proteins of diaphragm muscle fibers [6, 7]. In the absence of sedatives, reduced central respiratory drive is unlikely to explain reduced force output of the respiratory muscles in ICU patients [8]. Phrenic nerve neuropathy, as assessed by prolonged phrenic nerve conduction time, has been demonstrated in ICU patients, indicating that injury of the peripheral nerve may play a role in reduced force output [9].

**Fig. 1:**
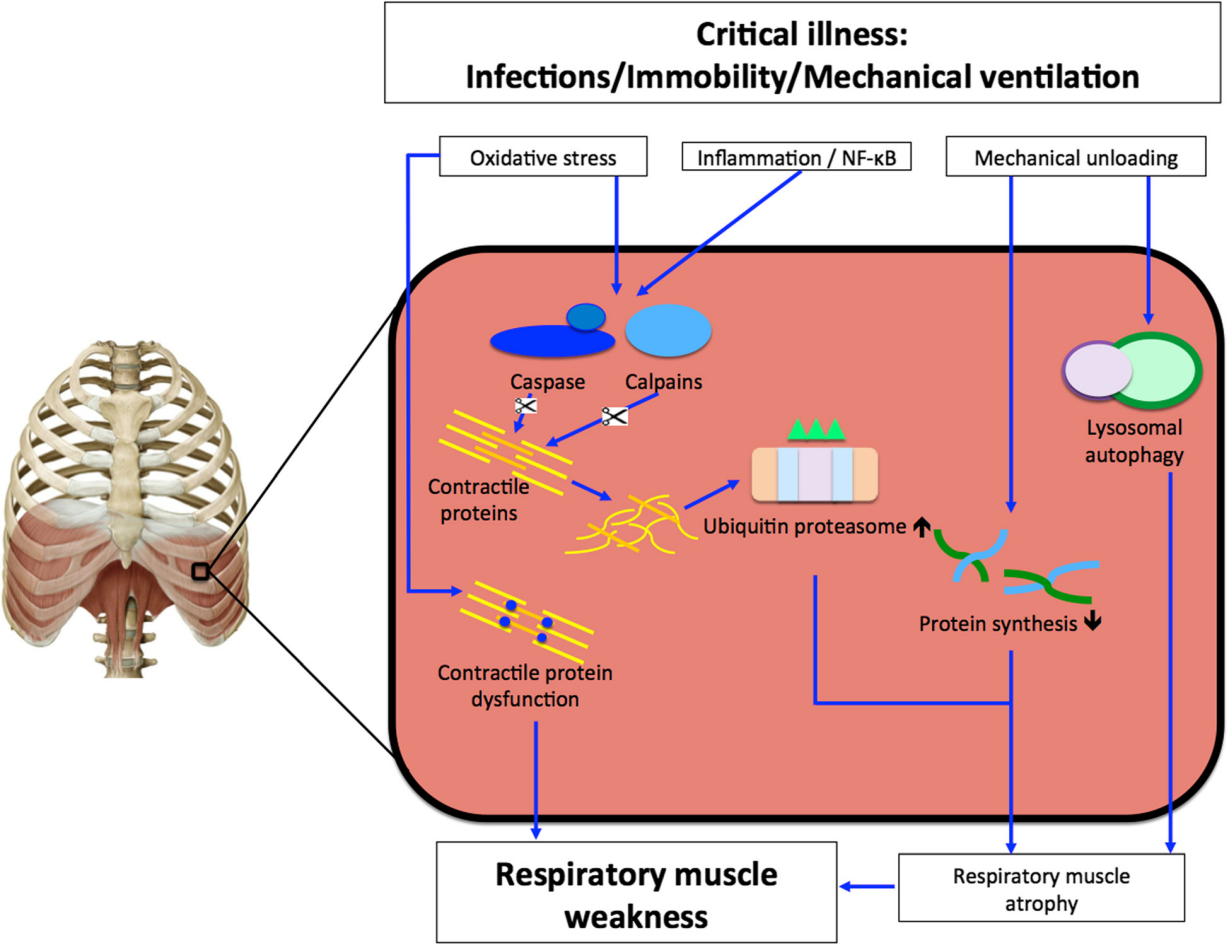
Proposed scheme of pathophysiologic pathways in the development of respiratory muscle weakness during critical illness. Oxidative stress [89], inflammation [71, 74], increased nuclear factor (NF)-κB activity [90], and mechanical unloading [10, 11] have been proposed to initiate respiratory muscle weakness. These initiators can result in contractile protein becoming dysfunctional [4], decreased synthesis [14, 15], or muscular autophagy [12]. Oxidative stress and inflammatory pathways can activate caspases and calpains [89, 91], thereby delivering substrates for the ubiquitin-proteasome [10, 11, 92], which further degrades contractile proteins

Contractile dysfunction of the respiratory muscles in ICU patients may result from the loss of muscle mass (atrophy) and/or dysfunction of the remaining contractile proteins. In a landmark paper, Levine and colleagues [10] demonstrated the rapid development of diaphragm muscle atrophy in ventilated brain-dead patients. More recently, Hooijman and colleagues [11] performed in-depth functional and structural analysis of diaphragm biopsies in critically ill patients on the ventilator. In that study, muscle fiber cross-sectional area was reduced by ±25 % after an average of 7 days of mechanical ventilation. Muscle atrophy is the final result of an imbalance between protein synthesis and degradation. Upregulation of several proteolytic pathways has been demonstrated in the respiratory muscles of ICU patients [11]. For instance, key regulators of the ubiquitin-proteasome pathway are upregulated in the diaphragm of these patients [10, 11]. Other pathways such as lysosomal protein degradation and autophagy may play a role as well (Fig. 1) [12, 13]. In addition to enhanced proteolysis, decreased protein synthesis has been reported in the diaphragm of rodents subjected to controlled mechanical ventilation [14, 15]. Besides atrophy, diaphragm weakness may be the result of contractile protein dysfunction. Even when corrected for loss of protein, muscle fibers in ICU patients develop less force [4]. Furthermore, the sensitivity of the contractile proteins for calcium is reduced [4]. The pathophysiology of contractile protein dysfunction in these patients is incompletely understood, but animal models of mechanical ventilation and endotoxemia indicate that phosphorylation and oxidative modifications of the sarcomeric proteins and mitochondrial proteins play a role in dysfunction and injury [16–19]. For an extensive background on the pathophysiology of muscle dysfunction in the critically ill, we refer to a recent excellent review on this subject [20].

## Evaluation of respiratory muscle function in ICU patients

We will briefly discuss the readily available techniques that are relevant and feasible in clinical practice. For a detailed overview we refer to other reviews [21–23]. Maximal inspiratory pressure (MIP) and maximal expiratory pressure (MEP) are used to evaluate global respiratory muscle strength and can be applied to selected ICU patients. MIP and MEP are measured using a handheld pressure device connected to the endotracheal tube or tracheostomy while the patient performs specific maneuvers. Although American Thoracic Society/European Respiratory Society guidelines advise a single inspiratory maneuver at residual volume in non-intubated patients [24], the reliability of MIP measurement in ICU patients is improved when using a unidirectional expiratory valve connected to the endotracheal tube or tracheostomy [25]. Alternatively, pressures can be assessed using the ventilator by performing an end-expiratory hold maneuver. A 20-s end-expiratory occlusion can be performed to obtain more reliable measurements in poorly cooperative patients [26]. It should be acknowledged that assessment of MIP and MEP requires a cooperative patient. Today, most ICU ventilators provide automatic functions to provide some useful parameters to evaluate the diaphragm function. High values for MIP and MEP exclude clinically significant weakness, but low values are common and may also reflect poor technique or effort [27].

Esophageal pressure (Pes) is an estimate of pleural pressure [28] and can be used to calculate the amount of work performed by the respiratory muscles. Simultaneous recording of Pes and gastric pressure (Pga) allows the calculation of transdiaphragmatic pressure (Pdi = Pga − Pes), a specific measure of diaphragm contractility. The latter is useful for close monitoring and evaluation of diaphragm function in difficult-to-wean patients [29]. However, acquisition, calculation, and interpretation of Pes, Pga, and their derived measures are rather complex and therefore not widely accepted in clinical practice.

Ultrasonography is an increasingly popular tool for assessment of diaphragm function in ICU patients [30]. In the subcostal view reduced caudal movement of the diaphragm during unassisted breathing is consistent with weakness and paradoxal movement indicates diaphragm paralysis [31, 32]. Diaphragm atrophy can be assessed by measuring diaphragm thickness in the midaxillary line at the level of the diaphragm dome [32, 33] and its thickening fraction during inspiration to asses contractile activity [30]. Diaphragm atrophy can also be evaluated more precisely with computed tomography, although this is more cumbersome than echography [34]. Using ultrasound, Goligher and colleagues [30] recently demonstrated in ICU patients that diaphragm atrophy is associated with diaphragm dysfunction and, in a minority of these patients, diaphragm thickness surprisingly increased while on the ventilator, which was associated with dysfunction as well. Diaphragm electromyography (EMG) reflects the electrical activity and is the gold standard to assess neural respiratory drive. Diaphragm EMG can be recorded best using an esophageal catheter with multiple electrodes. With the introduction of neurally adjusted ventilatory assist (NAVA; Maquet, Solna, Sweden) [35], the (processed) EMG signal can be obtained continuously in ICU patients. In these patients, diaphragm EMG may be used to monitor respiratory muscle unloading [36] and patient–ventilator interaction [37].

## Modulation of contractile activity: disuse and inspiratory muscle training

### Prevention of disuse atrophy

Like any other striated muscle, respiratory muscle mass is affected by contractile inactivity. In fact, the respiratory muscles appear more sensitive to the effects of disuse compared with other striated muscles [10, 11, 34]. In humans, relatively brief periods of diaphragm disuse (<3 days) due to controlled mechanical ventilation are associated with diaphragm muscle fiber atrophy [10].

Animal studies have demonstrated that mechanical ventilation-induced diaphragm atrophy and dysfunction is less severe in assisted modes of mechanical ventilation [38]. Recently, Goligher et al. [30] used ultrasound techniques to demonstrate that low levels of diaphragm activity, resulting from high levels of ventilator support, are associated with diaphragm atrophy and dysfunction in ICU patients. On the other hand, administration of muscle relaxants for 48 h in patients with early severe acute respiratory distress syndrome (ARDS) resulted in earlier withdrawal from mechanical ventilation and did not adversely affect peripheral muscle function [39]. Therefore, it appears that, under certain conditions (i.e., very severe ARDS), controlled mechanical ventilation is preferred to facilitate lung-protective ventilation and this beneficial effect outweighs the possible adverse effects on the respiratory muscles. Nevertheless, in general it is reasonable to limit the duration of controlled mechanical ventilation and prevent high levels of support under assisted ventilation in order to reduce the risk of disuse atrophy [10, 30, 40]. It is important to recognize that ventilator pressure and flow waveforms are unreliable to confirm the presence of respiratory muscle activity [21, 22, 41]. We recommend additional monitoring techniques as outlined above. Although the optimal level of activity for the respiratory muscles is unknown during mechanical ventilation, these monitoring techniques allow us to detect complete inactivity of the muscles due to over-assist.

### Inspiratory muscle training

In general, training can be instituted to enhance muscle endurance or strength. These types of training require different strategies and have distinct physiological responses. In healthy subjects, respiratory muscle activity is characterized by development of low pressure during the entire life span of a subject. The pressure generated by the inspiratory muscles is only ±5 cmH_2_O (5 % of maximum inspiratory pressure) to generate a tidal volume of 500 ml, when respiratory compliance is 100 ml/cmH_2_O. At first sight, training of the respiratory muscle should, therefore, be designed to improve endurance. Indeed, in patients who are difficult to wean from the ventilator, progressive weaning trials (T-tube or low pressure support) are frequently instituted as training stimulus. Although reasonable from a physiological perspective, it has never been proven that this strategy indeed improves respiratory muscle endurance.

In very few circumstances is high inspiratory pressure required for prolonged periods of time and therefore strength training of the diaphragm seems of limited relevance. It has been demonstrated, however, that respiratory effort sensation depends on the maximal inspiratory pressure [42]. In healthy subjects, pharmacological induction of inspiratory muscle weakness increases respiratory effort sensation for the same workload [42], confirming the importance of adequate strength beyond that strictly required to generate tidal volume. It has been shown that inspiratory muscle strength training (IMST) improves whole body exercise performance, in particular in less fit subjects [43]. Also, in patients with chronic obstructive pulmonary disease (COPD), IMST improves inspiratory muscle strength and total body exercise and reduces dyspnea sensation [44]. Only three randomized studies have reported the effectiveness of IMST in ventilated ICU patients. In the trial by Cader et al. [45], 41 ventilated ICU patients with respiratory muscle weakness were randomized between inspiratory threshold loading and no training intervention. Training consisted of an inspiratory load of 30 % maximum inspiratory pressure for 5 minutes, twice a day, 7 days a week throughout the weaning period. Maximum inspiratory pressure was significantly increased in the training group (15 ± 3 to 25 ± 4 cmH_2_O) but not in the control group (15 ± 2 to 18 ± 2 cmH_2_O). The study was underpowered for clinically relevant endpoints, although weaning time was reduced in the training group. In another study, Martin and colleagues [46] randomized 69 ventilator-bound patients (mean duration of ventilation at inclusion ±44 days) to IMST or sham training added to endurance training. Strength training consisted of four sets of 6–10 breaths per day with 2 minutes rest between each set. Loading was individualized and set at a level that could just be tolerated by the patient. IMST significantly improved maximum inspiratory pressure (end of training 54 cmH_2_O in intervention group and 45 cmH_2_O in sham group, *P* < 0.05). Also, successful weaning at day 28 after inclusion was more likely in the intervention group compared with sham (71 versus 47 %, *P* < 0.05). No adverse events of IMST were reported in these two trials. Finally, Condessa et al. [47] randomized ventilated patients to inspiratory strength training on top of usual care versus usual care only. Each training session consisted of five sets of loaded breaths (40 % maximum inspiratory pressure), twice a day, 7 days a week. In this study, IMST significantly increased maximum inspiratory pressure but did not affect weaning time.

In conclusion, inspiratory muscle training is feasible and appears safe in patients with respiratory muscle weakness who are difficult to wean from the ventilator, which is supported by a recent systematic review [48]. Studies in other patient categories, including COPD, indicate that IMST improves outcome. In our opinion, it is reasonable to add IMST to endurance training in stable, difficult-to-wean patients with confirmed respiratory muscle weakness. Future studies are needed to determine the optimal training protocol and appropriate timing for initiation of IMST.

## Antioxidants and nutrition

As outlined above, oxidative stress may play a role in the pathophysiology of ICU-acquired respiratory muscle weakness. Several experimental studies demonstrate that antioxidants attenuate the detrimental effects of controlled mechanical ventilation and/or systemic inflammation on respiratory muscle structure and function [18, 49–51]. In healthy subjects, high dose N-acetylcysteine (150 mg/kg intravenously) attenuated diaphragm fatigue induced by an inspiratory resistive load [52]. Today, no study has specifically evaluated the effect of antioxidants on respiratory muscle function in ICU patients; however, some indirect evidence is available. In an open trial, 595 critically ill surgery or trauma patients were randomized between antioxidant supplementation (alpha-tocopherol and ascorbic acid) and standard care or standard care only [53]. Patients in the antioxidant group spent less time on the ventilator (3.7 versus 4.6 days, *P* < 0.05). It should be noted that patients in this study were young (38 ± 15 years) and total ventilation time was rather short. Therefore, it is questionable whether the beneficial effects of antioxidants were the result of improved respiratory muscle function. Heyland et al. [54] studied the effects of antioxidants and glutamine in a heterogeneous ICU population (N = 1223). Patients were divided into four groups: placebo, glutamine (0.35 g/kg/24 h of body weight intravenously), antioxidants (selenium, zinc, beta carotene, vitamin E, and vitamin C), or antioxidants plus glutamine. Again, respiratory muscle function was not specifically evaluated but no difference in duration of mechanical ventilation was observed among the four groups.

Today, only one study has evaluated the effects of two nutritional strategies on skeletal muscle structure and function in critically ill patients [55]. This study was a planned subanalysis of the EPaNIC trial that compared the effects of tolerating macronutrient deficiency versus early parenteral nutrition on skeletal muscle structure and function [56]. In that study, patients were randomized between early (within 2 days of ICU admission) versus late (<8 days after ICU admission) parenteral nutrition to prevent macronutrient deficiency. Skeletal muscle strength was assessed in 600 ICU patients using the Medical Research Council sum score. Weakness occurred less often in the late versus early parenteral nutrition group (34 versus 43 %, *P* = 0.030). Compared with healthy subjects, muscle fibers exhibited atrophy but were not significantly different between the early and late parenteral nutrition groups. However, markers for autophagosome formation were significantly higher in the late parenteral nutrition group. This indicates that autophagy plays an important role in protein turnover next to other effects to provide substrate for recycling. In conclusion, tolerating macronutrient deficiency in the first week after ICU admission is not associated with the development of muscle fiber atrophy but surprisingly appears to improve muscle contractility.

Although the effect of high-dose antioxidant administration on respiratory muscle structure and function is encouraging in animal models, no data support the routine administration of antioxidants or other specific feeding strategies on respiratory muscle function in the critically ill ventilated patient.

## Improving respiratory muscle protein content: anabolics

Loss of muscle mass plays an important role in the development of ICU-acquired respiratory muscle weakness [10, 11]. Pharmacological interventions that restore protein balance seem a reasonable approach in these patients. First, inhibitors of proteolysis increase respiratory muscle mass in animal models with heart failure and under mechanical ventilation [57, 58] but toxicity limits the application of these agents for this specific indication in humans. Second, anabolic hormones have been used to enhance skeletal muscle mass under a variety of conditions (reviewed in [59]). We focus on the effects of endogenous and exogenous anabolic hormones on the respiratory muscles.

The most important endogenous anabolic hormones are growth hormone (GH), insulin-like growth factor-1 (IGF-1), insulin, and the anabolic steroid testosterone and its analogues. The effects of GH on skeletal muscle have been reviewed recently [60]. GH enhances production of IGF-1 in the liver but has direct anabolic effects on skeletal muscle as well. The effect of administration of GH/IGF-1 or its exogenous analogues on respiratory muscle function in critically ill patients has not been studied. However, Takala et al. [61] reported the effects of recombinant GH (Genotropin) administration in critically ill ICU patients relatively early during ICU stay in two placebo-controlled trials. Unexpectedly, both trials demonstrated significantly increased mortality in recombinant GH-treated patients (47 versus 25 % in the Finnish study and 61 versus 26 % in the multinational study). The mechanisms for increased mortality are incompletely understood, but modulation of immune function may play a role. Interestingly, Schols et al. [62] reported the effects of the exogenous anabolic steroid nadrolone in patients with stable COPD during an 8-week pulmonary rehabilitation program. Patients (N = 217) were randomized between placebo, placebo with high caloric feeding, and nandrolone with high caloric feeding. They found that nandrolone together with high caloric feeding significantly improved inspiratory muscle strength. These findings were more or less confirmed in a later trial by the same group [63]. Although case series report the use of anabolic steroids in difficult-to-wean patients [64], no randomized studies have evaluated the safety and efficacy of anabolic steroids in ICU patients with respiratory muscle weakness. Accordingly, anabolic hormones should not be used in the early stage of ICU admission but may have a role in more chronic and stable ICU patients with respiratory muscle weakness and who are difficult to wean from the ventilator.

## Positive inotropes

In addition to atrophy, dysfunction of the remaining muscle fibers has been demonstrated in critically ill patients [4]. Accordingly, optimizing contractility using positive inotropes seems a reasonable approach in these patients. ß-Adrenoreceptor agonists indeed exhibit a direct positive inotropic effect on the diaphragm muscle in vitro by increasing intracellular calcium influx. The effects of β2 adrenoreceptor agonists on respiratory muscle function in vivo are, however, controversial. Albuterol (oral) did not affect fatigability of the diaphragm in healthy subjects. However, fenoterol (oral) delayed the development of diaphragm fatigue in healthy volunteers subjected to inspiratory loading. In mechanically ventilated COPD patients with respiratory failure, respiratory muscle function significantly improved after dopamine infusion, probably by augmentation of diaphragm blood flow and improved cardiac output. Currently the administration of ß-adrenoreceptor agonists cannot be recommended to improve respiratory muscle function in ICU patients.

Muscle fibers isolated from the diaphragm of ICU patients display decreased maximal force-generating capacity, indicating intrinsic muscle weakness [11]. In fast-twitch diaphragm fibers the reduction of sub-maximal force generation even exceeds the loss of maximal force-generating capacity [4]. This implies that these fibers require more calcium to generate normal force levels, i.e., their sensitivity to calcium is reduced. Calcium sensitizers have been developed to treat similar pathology of cardiac muscle [65, 66]. Currently, levosimendan is the only calcium sensitizer approved for use in humans (>50 countries worldwide). Experimental studies have shown that levosimendan improves calcium sensitivity of diaphragm muscle fibers from patients with COPD [67]. Moreover, a recent double-blind, randomized, crossover study demonstrated that administration of levosimendan improved neuromechanical efficiency by >20 % and prevented contractile fatigue during a diaphragm-loading task in healthy subjects [68]. A randomized clinical trial (ClinicalTrials.gov identifier NCT01721434) is currently investigating whether levosimendan indeed facilitates liberation from the ventilator. In contrast to levosimendan, the effectiveness of other calcium sensitizers has so far only been studied in vitro. For example, exposure to EMD 57033 (a troponin activator) partially restored calcium sensitivity in diaphragm fibers isolated from piglets after 5 days of mechanical ventilation [69]. Furthermore, in diaphragm fibers from critically ill patients, CK-2066260 completely restores calcium sensitivity [4].

Taken together, calcium sensitizers might exert an energetically beneficial effect on diaphragm work [67]. When less calcium is needed to maintain force generation, muscle work becomes more efficient [68] and could improve respiratory muscle contractility in critically ill patients. However, further clinical trials are needed to prove the benefits of the calcium sensitizers in ICU patients with respiratory muscle weakness.

## Future developments

### Modulation of inflammation

Activation of pro-inflammatory pathways is associated with respiratory muscle weakness [70–73]. Therefore, modulation of the inflammatory response to combat respiratory muscle dysfunction has been a focus of interest in recent experimental studies [71, 74, 75]. We demonstrated, for instance, that interleukin (IL)-6 plays an important role in the loss of contractile proteins in muscle fibers exposed to plasma from septic shock patients [71]. However, diaphragm fiber atrophy due to disuse in brain death patients was not associated with upregulation of IL-6 [10]. IL-10 is an interleukin with anti-inflammatory properties [76]. In a murine model of *Pseudomonas aeruginosa* pneumonia, diaphragm dysfunction was attenuated after experimental IL-10 administration [76]. During critical illness with subsequent inflammatory status, nuclear factor (NF)-κB is the key factor for transcription of several cytokines [77]. Recently, evidence was found that inhibition of NF-κB in endotoxemic mice protects against diaphragm muscle weakness, probably due to decreased generation of pro-inflammatory cytokines [75]. Proteolytic pathways can be activated through Toll-like receptor (TLR)-4, present in muscle plasma membrane [74]. TLRs are essential receptors in recognizing microbes and initiating an inflammatory immune response [78]. In TLR-4 knockout mice, loss of diaphragm contractile protein associated with controlled mechanical ventilation was attenuated compared with wild-type mice [74]. In a large clinical trial the TLR-4 antagonist eritoran did not, however, improve outcome in patients with severe sepsis or septic shock (±80 % on mechanical ventilation) [79]. Nevertheless, neither skeletal muscle function nor duration of mechanical ventilation was assessed in this trial.

Traditionally, steroids are associated with myopathy, atrophy, and dysfunction of the respiratory muscles [80–82]. However, the final effects appear to be dose- and time-dependent, at least in experimental studies. For instance, Maes et al. [83] demonstrated that “low-dose” (5 mg/kg) methylprednisolone exacerbated ventilator-induced diaphragm dysfunction in rats, whereas a high dose (30 mg/kg) protected against the deleterious effects of controlled mechanical ventilation on diaphragm function. In ICU patients the effect of steroids on muscle function are conflicting [84–86]. However, no study has prospectively evaluated the effects of corticosteroids on respiratory muscle function in ventilated ICU patients.

In conclusion, despite the encouraging data that modulation of inflammation improves respiratory muscle function in animals, data in humans are scarce and, where present, disappointing.

### Modulation of proteolytic pathways

Since activation of proteolytic systems plays a key role in the development of respiratory muscle dysfunction during critical illness, several experimental studies have investigated the effect of specific inhibitors. For instance, in rats exposed to 24 h of mechanical ventilation, bortezomib treatment partially prevented the reduction of diaphragm force and atrophy [58]. These small positive effects were probably mediated by the ability of bortezomib to indirectly reduce caspase-3 activity [57]. Proteasomes are only able to process myofilaments that have been cleaved from the sarcomere by enzymes like caspases and calpains [87]. Accordingly, inhibition of the proteasome alone is not expected to have substantial effects on muscle function as this would leave the muscle cell with only unprocessed, but cleaved, myofilaments. Furthermore, considering the basic housekeeping cell functions of the proteasome, it is no surprise that the clinical application of bortezomib is accompanied by serious toxic adverse events, such as cytopenia and peripheral neuropathy [88]. Finally, although several compounds targeting proteolytic pathways upstream of the proteasome have a high potential to prevent the development of diaphragm weakness, this does not necessarily imply that these agents can also improve function of the weakened diaphragm. Nevertheless, modulation of the proteolytic system is a potentially interesting target to modulate loss of respiratory muscle function due to controlled mechanical ventilation.

## Conclusion

**Fig. 2:**
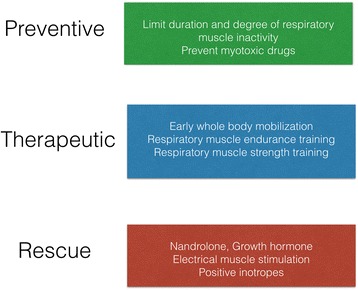
Three groups of interventions to counteract respiratory muscle weakness during critical illness

Weakness of the respiratory muscles frequently develops in the ICU patient and is associated with adverse outcome, including prolonged mechanical ventilation. Despite the high incidence and clinical impact of ICU-acquired respiratory muscle dysfunction, no specific preventive or therapeutic interventions have been tested in large randomized controlled trials. Therefore, we should rely on interventions that seem reasonable from a physiological perspective or are supported by small clinical studies. As pointed out in Fig. 2, interventions could be subdivided into three categories: prevention of respiratory muscle dysfunction; therapeutic strategies that aim to improve respiratory muscle function; and so-called rescue interventions that should only be applied in exceptional cases and only after discussion with the patient or primary decision makers.

Preventive strategies should limit development of disuse atrophy and muscle damage. We suggest using techniques that monitor diaphragm muscle function [21, 22] to confirm a physiologically acceptable level of diaphragm contractility and allow the clinician to optimize ventilator settings in order to improve patient–ventilator interaction. Drugs with potential side effects on skeletal muscle, in particular corticosteroids and muscle relaxants, should be avoided when appropriate.

Once ICU-acquired weakness has developed, a combined program of respiratory muscle endurance training and strength training should be considered. Endurance training can be instituted using progressive weaning trials and strength training by using a device for variable inspiratory threshold loading connected to the endotracheal tube [46]. Use of respiratory muscle positive inotropes, in particular levosimendan, is the subject of a current randomized controlled trial (NCT01721434) and not currently recommended for difficult-to-wean patients.

## Abbreviations

ARDS: acute respiratory distress syndrome
COPD: chronic obstructive pulmonary disease
EMG: electromyography
GH: growth hormone
ICU: intensive care unit
IGF-1: insulin-like growth factor-1
IL: interleukin
IMST: inspiratory muscle strength training
MEP: maximal expiratory pressure
MIP: maximal inspiratory pressure
NF: nuclear factor
Pdi: transdiaphragmatic pressure
Pes: esophageal pressure
Pga: gastric pressure
TLR: toll-like receptor
